# Introduction to the Special Issue on Molecular Guided Surgery

**DOI:** 10.1117/1.JBO.30.S1.S13701

**Published:** 2025-05-07

**Authors:** Brian W. Pogue, Sylvain Gioux, Summer L. Gibbs

**Affiliations:** a University of Wisconsin School of Medicine & Public Health, Department of Medical Physics, Madison, Wisconsin, United States; b Intuitive Surgical, Aubonne, Switzerland; c Oregon Health & Science University, Biomedical Engineering Department, Portland, Oregon, United States; d Oregon Health & Science University, Knight Cancer Institute, Portland, Oregon, United States

## Abstract

The editorial completes the Special Issue on Molecular Guided Surgery for Volume 30 of the Journal of Biomedical Optics.

This special issue on molecular guided surgery (MGS) was designed to highlight the 10-year anniversary of the SPIE BiOS conference stream with the same focus. The conference on MGS was started in 2014 by Dr. Sylvain Gioux (University of Strasbourg, France; now at Intuitive Surgical) and Professor Brian Pogue (Dartmouth, USA; now at University of Wisconsin–Madison) to address the need to bring together like-minded researchers who were shaping the emergent field of surgical guidance^1^^,^^2^ with different molecular contrast tools in several different surgical subspecialties.^3^^–^^5^ The SPIE BiOS conference became an ideal place to co-locate researchers who were advancing these tools and techniques in the field,^6^ and also to bring together translational physicians who were willing to engage with the technical innovators. Most importantly the conference always welcomed the participation and sponsorship of entrepreneurial and established companies who were advancing this MGS space as well. The latest version of the conference is now chaired by Professor Summer Gibbs (Oregon Health & Science University) and Professor Kenneth Tichauer (Illinois Institute of Technology).

The mix of expertise in the conference has involved synthetic chemistry, medicinal chemistry, biological targeting, cellular and molecular contrast agents, tissue optics, device development, software development, early phase rodent studies, and all the way to translational studies involving large animals and humans. At the time of founding in 2014, there were over 1000 peer-reviewed papers being published per year in PubMed, but there was not an ideal technical venue for presentation and discussion. Today this has grown to be over 2000 peer-reviewed papers in PubMed, and well over a dozen successful companies. Many of the earliest entrepreneurial companies in this field were acquired by larger surgical device conglomerates, a sign of adoption into the mainstream surgical device marketplace. Additionally, while the use of indocyanine green (ICG) had and continues to dominate the applications in this field, the regulatory approval and clearance of several metabolic and immuno-targeted agents has occurred in many countries, widening the use case of molecular guided surgery.

At the start of the MGS conference, it was decided that it would be heavily weighted to invited speakers who had accomplished major advancements in the field, and also a diversity of science that spread across the areas of endogenous molecular contrast, exogenous molecular contrast, developing contrast agents, and clinical translation. Importantly it was decided early on that endogenous and exogenous contrast mechanisms should be equally represented so as not to discount the idea that advances in instrumentation might lead to improved label-free imaging with molecular contrast, with techniques such as Raman, infrared (IR), vibrational, terahertz, speckle, coherence, spatial frequency, and other hardware designs for imaging.^7^^–^^9^ Key invited speakers were found, headlined by Dr. Bruce Tromberg, PhD, as head of the Beckman Laser Institute (now director of the National Institute of Biomedical Imaging and Bioengineering). The conference progressed from 1 day in 2014 to 2 days in 2015, with submitted abstracts matching the invited speakers, to round out the program. The program was ultimately always constrained to the 2 weekend days of the BiOS meeting to allow for a focused time, without extending it beyond what was viewed as a useful limit.

This special issue for the *Journal of Biomedical Optics* (JBO) draws from invited contributors to the conference from 2024, with a cross-section of papers that cover the fields and areas of focus of the conference. There are studies that validate and characterize MGS features of tissue or contrast agents, and studies that show improved imaging systems through multi-wavelength band imaging, and finally studies demonstrating software and hardware advancements that make imaging systems amplify the contrast that is present.

The first characterization paper, by Wang and colleagues based in Fujian Medical Center (China) and Massachusetts General Hospital (USA),^10^ reports a study investigating the autofluorescence properties of parathyroid tissue using near-infrared light with both fresh and paraffin-embedded thyroidectomy specimens. The relevance of this work extends to the emerging field of parathyroid gland identification during fluorescence guided thyroid surgeries, potentially reducing the risk of accidental removal or damage to these glands. The second paper, by Kriukova and colleagues at multiple institutions,^11^ reports a benchmarking study based upon the observed signal-to-noise ratio (SNR) and contrast definition from fluorescence molecular imaging systems. The research highlights the importance of standardizing methods to key measurable parameters, that ultimately will enhance the quality and reliability of fluorescence imaging. The third paper, by Scorzo and colleagues at Dartmouth,^12^ compares spatial distributions of two clinically used fluorophores, ALA-PpIX and ICG, in a model glioma, using 3D fluorescence cryotomography techniques. This study demonstrated how to achieve an *ex vivo* analysis of the *in vivo* distribution for two of the most-used fluorescent contrast agents in glioma tumor resection, while using the more realistic size glioma tissue model in swine. The fourth paper, by Sever and colleagues based at the University of Pittsburg,^13^ is about dual imaging with gamma detection and IR fluorescence, using Indium-111 and IRDye800CW to compare the use of gamma and IR as intraoperative molecular imaging tools. This was a detailed tissue phantom model study that helps to inform the depths of sensitivity for the two techniques.

A series of papers were contributed on hardware innovations that combine imaging at multiple wavelength bands or multiple agents. The first such paper, by Szafran and colleagues at Oregon Health & Science University and at Dartmouth, demonstrates dual-color fluorescence-guided surgery for head and neck cancer resection (featured on the issue cover) and investigates an experimental but important technique to improve the specificity of imaging of the reporters.^14^ The next paper, by Keizers and colleagues, was a collaboration across the University Medical Center in Groningen, with two companies and Helmholz Zentrum Munich, and is a systematic comparison of fluorescence imaging between near-infrared and shortwave-infrared spectral ranges using clinical tumor samples.^15^ The findings provide insights into the advantages and limitations of each spectral range, guiding the selection of appropriate imaging techniques for future clinical applications.^15^ The paper by Kulkarni and colleagues at University of Wisconsin^16^ showed that it is possible to design a dual-channel fluorescence depth-sensing system for simultaneous measurement of ICG and PpIX, where the two emissions are captured at the same time in two detectors while still being able to normalize the data by the transmitted excitation at each excitation wavelength. This system was demonstrated in use through experimental tumors growth sensing *in vivo*.^16^

Two papers were contributed in the area of augmenting the contrast visible through algorithms and careful sensing with structured light. A paper by Won and colleagues at the University Health Network in Toronto, Canada,^17^ discusses the use of deep learning algorithms to enhance value in surgical guidance, specifically for fluorescence imaging in oral cancer depth quantification. This combination of the latest artificial intelligence methods with imaging technologies is a critically important sector to highlight. A second paper, by Song and colleagues at Johns Hopkins University,^18^ demonstrates a design for speckle illumination for spatial frequency domain imaging through a stereo laparoscope for endogenous contrast enhancement during minimally invasive surgery.

Lastly, a paper by Nguyen, Torres, and colleagues at Dartmouth and at Cedars-Senai Medical Center reviews recent pre-clinical and clinical studies that use fluorescence molecular imaging to assess surgical margins, highlighting the most promising fluorescence imaging technologies currently utilized in the surgical suite and laboratory for peripheral and deep *en face* margin assessment, as well as a proposed margin assessment platform (“MAP”) that incorporates both macroscopic and, meso- or microscopic imaging with post-processing and machine learning.^19^

The MGS field continues to advance and evolve as new agents are discovered and developed, new imaging systems are invented, and algorithms are refined to advance their capabilities. The translational work has been in both directions, with bench-to-bedside trials started up, and a large number of now clinical to basic research investigative studies to determine what is being seen. The conference continues at the SPIE BiOS meeting at Photonics West every January in San Francisco, with ample time for exchange and discussion of ideas.
